# A Comprehensive Analysis Using Colorimetry, Liquid Chromatography-Tandem Mass Spectrometry and Bioassays for the Assessment of Indole Related Compounds Produced by Endophytes of Selected Wheat Cultivars

**DOI:** 10.3390/molecules26051394

**Published:** 2021-03-05

**Authors:** Agnieszka Kuźniar, Kinga Włodarczyk, Ilona Sadok, Magdalena Staniszewska, Małgorzata Woźniak, Karolina Furtak, Jarosław Grządziel, Anna Gałązka, Ewa Skórzyńska-Polit, Agnieszka Wolińska

**Affiliations:** 1 Department of Biology and Biotechnology of Microorganisms, The John Paul II Catholic University of Lublin, 1I Konstantynów Str., 20-708 Lublin, Poland; kinga.wlodarczyk@kul.pl (K.W.); agnieszka.wolinska@kul.pl (A.W.); 2 Laboratory of Separation and Spectroscopic Method Applications, Centre for Interdisciplinary Research, The John Paul II Catholic University of Lublin, 1J Konstantynów Str., 20-708 Lublin, Poland; ilona.sadok@kul.pl (I.S.); magdalena.staniszewska@kul.pl (M.S.); 3 Institute of Soil Science and Plant Cultivation State Research Institute, 8 Czartoryskich Str., 24-100 Puławy, Poland; m.wozniak@iung.pulawy.pl (M.W.); kfurtak@iung.pulawy.pl (K.F.); grzadziel@iung.pulawy.pl (J.G.); agalazka@iung.pulawy.pl (A.G.); 4 Department of Plant Physiology and Biotechnology, The John Paul II Catholic University of Lublin, 1I Konstantynów Str., 20-708 Lublin, Poland; eskorzynska@kul.pl

**Keywords:** indole-3-acetic acid, indole related compounds, endophytic bacteria, wheat, auxins, liquid chromatography-mass spectrometry

## Abstract

Liquid chromatography–tandem mass spectrometry (LC–MS/MS)**,** colorimetry, and bioassays were employed for the evaluation of the ability of endophytic bacterial strains to synthesize indole-related compounds (IRCs) and in particular indole-3-acetic acid (IAA). A total of 54 endophytic strains belonging to seven bacterial genera isolated from tissues of common and spelt wheat cultivars were studied. The endophytic bacteria isolated from different tissues of the tested wheat types were capable of IRCs production, including IAA, which constituted from 1.75% to 52.68% of all IRCs, in in vitro conditions via the tryptophan dependent pathway. The selected post-culture medium was also examined using a plant bioassay. Substantial growth of wheat coleoptile segments treated with the bacterial post-culture medium was observed in several cases. Our data suggest that the studied endophytic bacteria produce auxin-type compounds to support plant development. Summarizing, our approach to use three complementary methods for estimation of IRCs in different endophytic strains provides a comprehensive picture of their effect on wheat growth.

## 1. Introduction

Plants need appropriate environmental conditions such as light, water availability, mineral nutrients, etc. for proper growth and functioning. However, their growth and development is also regulated by phytohormones [1]. This process can be modulated by biotic factors, e.g., microorganisms that play a key role in symbiotic relations with plants. The symbiosis is known to increase the resistance of plants in a wide variety of environmental conditions [2,3]. Importantly, distinctive communities of microorganisms can modify the ecological niche in which plants grow. They can also directly affect the metabolic state of plants and participate in the biological protection thereof [2].

One such group of microorganisms with a beneficial effect on plants are endophytes (bacteria and fungi colonizing the host plant throughout the entire or part of the life cycle without inducing signs of disease in plant tissues) [4]. Thanks to the symbiotic relationship, the endophyte gains access to freely accessible nutrients. In turn, plants are offered an extra supply of beneficial nutrients, some secondary metabolites, and enzymes [2]. In general, endophytic microorganisms can improve plant efficiency via many different mechanisms [3,5]. They facilitate the uptake and accumulation of minerals and nutrients such as phosphorus (P) or nitrogen (N). They can also protect plants from pathogens, improve soil structure, and degrade pollutants [6]. A very important function of endophytes is the direct or indirect stimulation of plant growth through secretion of enzymes and phytohormones [7]. In addition, endophytes influence plant’s hormonal balance [8].

Phytohormones are small signaling molecules with a diverse chemical structure [7] controlling all aspects of plant growth and development during ontogenesis [8,9]. They can be synthesized in other cells and transported to tissues where there is the greatest demand for these phytohormones. Some hormones present in plants, such as auxins, cytokinins, gibberellins, salicylic acid (SA), jasmonic acid (JA), and abscisic acid (ABA), are produced by bacterial endophytes. It is worth mentioning that auxins are fundamental substances in the plant life cycle [7]. They positively influence cell division and enlargement, tissue differentiation, apical dominance, bud formation, and root initiation. Moreover, auxins promote the production of other hormones and control the growth of stems, roots, and fruits [10]. Auxins naturally occur in the active forms as indole-3-acetic acid (IAA), 4-chloroindole-3-acetic acid (4-Cl-IAA), and phenylacetic acid (PAA); inactive precursors: indole-3-pyruvic acid (IPyA), indoleacetamine (IAM), indole-3-acetaldoxime (IAOx), indole-3-acetonitrile (IAN), and indole-3-acetaldehyde (IAAld); and storage forms: indole-3-butyric acid (IBA), methyl-IAA (MeIAA), and auxins conjugated to amino acids or sugars [11,12]. However, it should be pointed out that high auxin concentrations have inhibitory activity; hence, the endogenous level must be constantly controlled by plants. The two main pathways: tryptophan (Trp)-independent and Trp-dependent pathways for IAA biosynthesis have been recognized in plants [5,13]. Trp is synthesized from chorismate in the chloroplast and then used as a substrate in the Trp-dependent IAA pathway. This biosynthesis can occur in four modes: (i) the indole-3-acetamide (IAM) pathway, (ii) the indole-3-pyruvic acid (IPA) pathway, (iii) the tryptamine (TAM) pathway, and (iv) the indole-3-acetaldoxime (IAOX) pathway. In Trp-independent IAA biosynthesis, indole-3-glycerol phosphate or indole act as precursors [13]. Plants can use some mechanisms to control the level of IAA, e.g., biosynthesis, degradation, transport, and conjugate formation [5].

Important is the fact that endogenous IAA in plants mostly occurs in a conjugated form that remains biologically inactive. IAA can be conjugated to monosaccharides, high molecular weight polysaccharides, myoinositol, choline, and carbohydrate components of glycoproteins via ester bonds. Moreover, IAA can also be conjugated to single amino acids, peptides, or proteins via amide bonds [14]. The IAA pathway via IAM is considered as a bacterium-specific mode. Additionally, most endophytes utilize L-tryptophan, which is secreted in root exudates as a precursor for IAA production [10].

Several studies have been carried out to confirm that endophytic bacteria isolated from wheat, i.e., *Bacillus altitudinis* WR10 originating from the roots of winter wheat *Triticum aestivum* L. Zhoumai 26, was capable of synthesizing IAA in amounts that were able to stimulate plant growth [15]. From sterilized roots and stems of *T. aestivum* cv. Chinese Spring, *T. aestivum* cv. PBW 343, and *T. aestivum* cv. HD 2967, Rana et al. isolated 159 endophytic bacteria that were screened in vitro for plant growth-promoting attributes. They confirmed that only five isolates were able to produce IAA [16] and, among them, *Acinetobacter guillouiae* EU-B2RT.R1 was found to be capable of IAA synthesis both in the presence (11.40 ± 0.00 mg·L^−1^) and absence (13.6 ± 0.01 mg·L^−1^) of tryptophan. Kiani et al. studied root, stem, and leaf tissue of winter wheat cultivars NARC 2011, Ujala 2015, TW1410, Inqilab 91, Galaxy 2013, and 15BT023 and found 10 isolates with a capability of IAA production [17]. However, there is no knowledge about the ability to synthesize IAA in leaves, roots, and coleoptiles of *T. aestivum* L. cv. ’Hondia’, *T. aestivum* L. cv. ’Tytanika’, and *T. spelta* L. cv. ’Rokosz’.

Given the important features and functions of IAA mentioned above, we hypothesized in the current study that selected endophytic bacterial strains isolated from the tissues of winter wheat cultivars (Hondia and Tytanika) and spelled wheat (Rokosz cultivar), which are common in Poland, might be able to produce biologically active forms of IAA. Consequently, the main goal of the study was to confirm the efficiency of biologically active IAA production by selected endophytes present in *T. aestivum* L. and *T. spelta* L. tissues using three independent techniques: colorimetric, liquid chromatography–tandem mass spectrometry (LC-MS/MS), and biological test (biotest).

## 2. Results

### 2.1. Identification of Endophytes

The molecular identification of the bacterial endophytes was carried out by the comparative sequence analyses of the standard bacterial sequence marker 16S rDNA. With the use of an interactive tool Blast n and NCBI database, we were able to identify all isolates at the genus level. The studied strains belonged to seven genera, including *Bacillus* (46), *Serratia* (3), *Lysinibacillus* (2), *Paenibacillus* (2), and *Pantoea* (1). A majority of the endophytes originated from leaves (24), followed by roots (17) and coleoptiles (13). In terms of the wheat cultivars, it was noted that Tytanika seemed to exhibit the highest richness of endophytes (25 isolates), whereas Rokosz was the least extensively colonized cultivar (11 isolates). Eighteen isolates originated from the Hondia cultivar. The summary of all isolates and their accession numbers in the GenBank database are listed in Table A1 (Appendix A).

### 2.2. Screening of Production of Indole Related Compounds (IRCs) by Endophytic Isolates—Colorimetric Method

**Figure 1:**
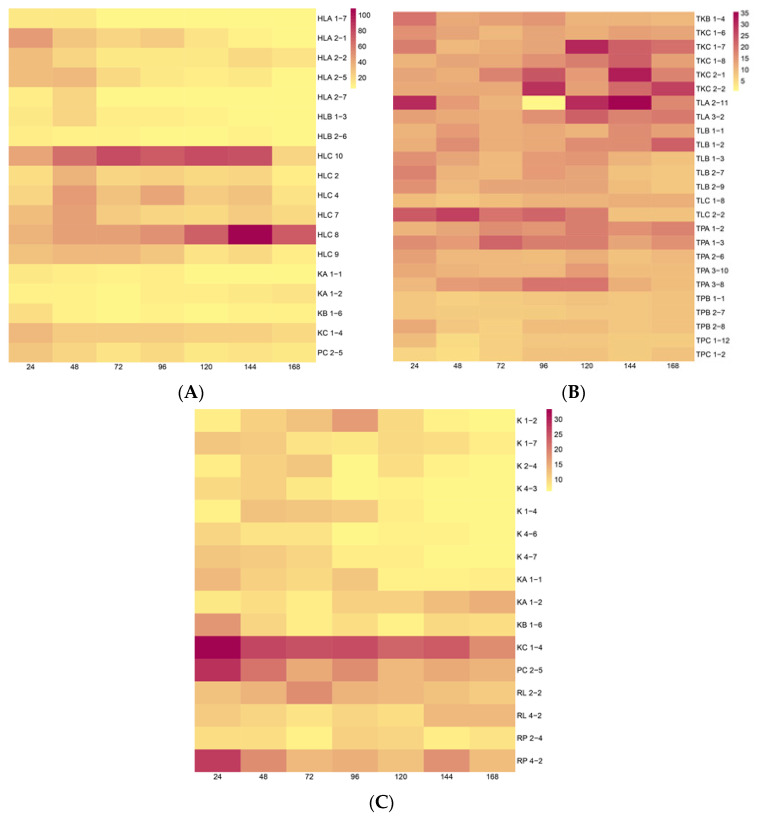
Heatmap of the amount of IRCs (µg∙mL^−^^1^) estimated using the Salkowski reagent in a medium collected every 24 h from bacterial cultures isolated from: (**A**) *T. aestivum* L. cv. Hondia, (**B**) *T. aestivum* L. cv. Tytanika, (**C**) *T. spelta* L. cv. Rokosz grown with 5 mmol∙ L^−1^ L-tryptophan. The figure presents the background subtracted concentrations obtained after subtraction of the amount in the sterile nutritional broth from the average concentration in the sample calculated from the standard curve. The mean and standard deviation were calculated from 3 biological replicates.

**Figure A1:**
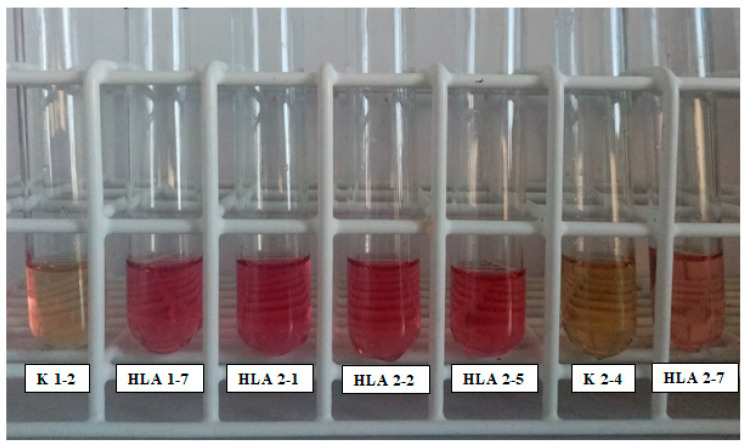

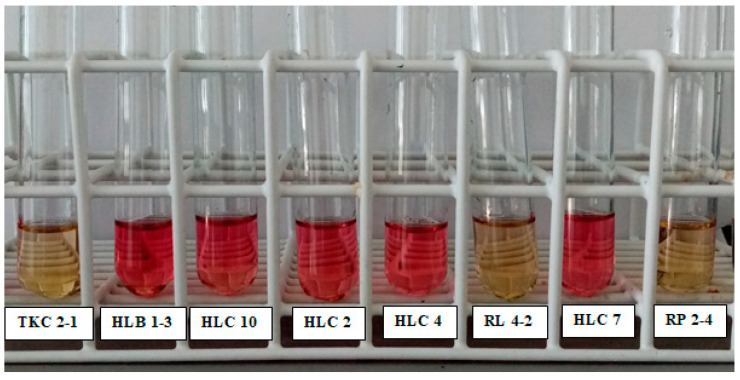
Examples of results of colorimetric analysis of IRC from different strains.

Our study was performed using the Salkowski reagent for IRC measurements at a maximum absorbance (530 nm) resulting in a pinkish solution. The concentrations of IRCs were estimated using an IAA standard curve (y = 0.0168x + 0.0896; R^2^ = 0.99). The colorimetric test demonstrated a varied ability of the endophytic bacteria isolated from the different wheat species to synthesize IRCs in the tryptophan (Trp) pathway. The results evidenced that 18 isolates from Hondia were able to produce IRCs, which was confirmed by the pink color of the sample (Figure A1, Appendix A). Our study also indicated that 25 of the tested bacterial strains originating from *T. aestivum* L. cv. Tytanika were positive in the Salkowski reagent test (pink color). All of the 11 isolates from *T. spelta* L. cv. Rokosz displayed a pink color with the use of the colorimetric assay, suggesting the presence of IRCs in the post culture medium (Figure A1, Appendix A). As a positive control, we applied the Salkowski assay to *Micrococcus* sp. AzoEndo14, which are well-studied IAA-producing plant growth-promoting bacteria (PGPB). The heatmap in Figure 1 shows the amount of IRCs in the bacterial culture media grown with 5 mmol∙L^−1^ l-tryptophan.

We observed different rates and kinetics of IRC production. The highest IRC yield (108.26 μg∙mL^−1^) was recorded for the endophytic strain (HLC 8) isolated from the leaves of *T. aestivum* L. cv. Hondia after 144 h (Figure 1). In contrast, the lowest rates of IRC synthesis (9.52–16.69 µg∙mL^−1^) was noted for isolates (K 4-3 and K 1-2) originating from the roots of *T. spelta* L. cv. Rokosz. The amount of IRCs released by the endophytes to the medium varied depending on the time of culturing. It was found that 10 strains were able to secrete IRCs continuously in their culture medium in shaking culture for up to 168 h. These strains were marked with the following codes: HLC 10, HLC 2, HLC 4, HLC 7, HLC 8, HLC 9, HLA 2-2, HLA 2-5, RP 4-2, and TPA 1-2 (Table A1, Figure 1). In contrast, selective production of IRCs by the tested strains was also evidenced by confirmation of the presence of IRCs in only one-day cultivation treatment. This trend was verified in strains with the following codes: TPC 1-2, TPC 1-12, RL 4-2, K 2-4, and K 1-7 (Figure 1C).

### 2.3. Determination of Indole-3-Acetic Acid by LC-MS/MS

**Figure 2:**
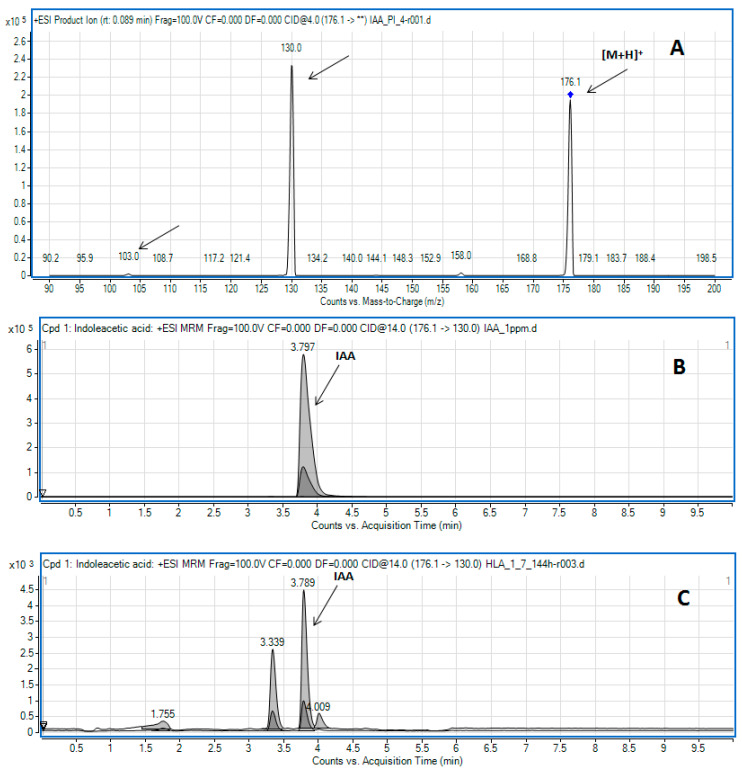
(**A**) Product ion mass spectra of protonated IAA (5.71 µmol L^−1^) recorded by flow injection analysis. Ions selected for MRM are marked with arrows. (**B**,**C**) Example of MRM results obtained during LC-MS/MS analysis of the IAA standard solution (**B**,**C**) bacterial culture supernatant of strain HLA 1-7 after 144 h of culturing.

The LC-MS/MS method was applied to confirm the presence of IAA among IRCs in the post-culture medium. In the chromatographic conditions applied, in the positive ion spectrum, free IAA shows an ion at *m*/*z* 176.1 corresponding to the protonated molecule ([M + H]^+^) [18]. Its fragmentation results in quinolinium ions at *m*/*z* 130.0 and much less intensive ions at *m*/*z* 103.0 (Figure 2A). Based on these observations, the transitions 176.1 > 130.0 (quantifier) and 176.1 > 103.0 (qualifier) were monitored in the multiple reaction monitoring (MRM) mode (Figure 2B,C) to determine IAA generated by the studied bacterial species.

The quantitative analysis of free IAA was done based on the matrix-matched calibration curve. The calculated limits of determination (LOD) and quantification (LOQ) for IAA were 0.002 and 0.007 µg∙mL^−1^, respectively. The calibration plot was linear over the IAA concentration range of 0.010–1.995 µg∙mL^−1^. The mean regression equation was *y* = 2413.9*x* − 324.6 (the coefficient of determination, R^2^ = 0.9953). The standard deviation for the slope and intercept were ±14.36 and 9.96 (*n* = 3), respectively.

Is it worth mentioning that the LC-MS/MS analysis was employed for samples that gave both positive and negative results in the Salkowski assay (Table 1). Examples of LC-MS/MS results obtained for free IAA determined in the standard solution and bacterial post-culture medium are presented in Figure 2B,C, respectively. Since the LC-MS/MS method is more sensitive, it was possible to determine IAA in samples where the analyte was undetected with the Salkowski method. For example, in the medium from the HLA 1-7 strain, the production of IAA was confirmed after 120 h of culturing, as evidenced by the pink color after the addition of the Salkowski reagent [19]. While there were no IRCs detected with Salkowski reagent at 144 h of culturing, the LC-MS/MS analysis evidenced correspondence of the IAA peak in the crude bacterial supernatant, as illustrated in Figure 2C and Table 2.

The trend of no positive results in the colorimetric method and the presence of a low concentration of IAA was confirmed by the LC-MS/MS method in several other cases, i.e., strains HLA 2-7, HLB 1-3, HLB 2-6, K 1-4, PC 2-5, RL 2-2, RP 2-4, TPA 1-4, and TKC 2-1. The summary of the results is shown in Table 3. Moreover, we did not observe any correlation with the tissues from which they were isolated or the cultivation time. Our results showed generation of 0.301 µg∙mL^−1^ to 11.770 µg∙mL^−1^ of IAA by the tested endophytic strains.

### 2.4. Biotest of Selected Crude Bacterial Supernatants

The biological activities of IRCs were tested for the selected endophyte strains cultivated in in vitro conditions (Table 2).

After 24 h of culturing, the IRCs/IAA secreted into the medium by the examined endophytes resulted in a slight increase in the growth of coleoptile segments; after 48 h, the growth was lower by 3.66–10.2% in comparison to the control. The PC 2-5 and KC 1-4 strains secreted over 2.789 µg∙mL^−1^ IAA into the medium (measured by LC-MS/MS), which was correlated with stronger growth stimulation of coleoptile segments (by 14–18%) than in the control.

**Figure 3:**
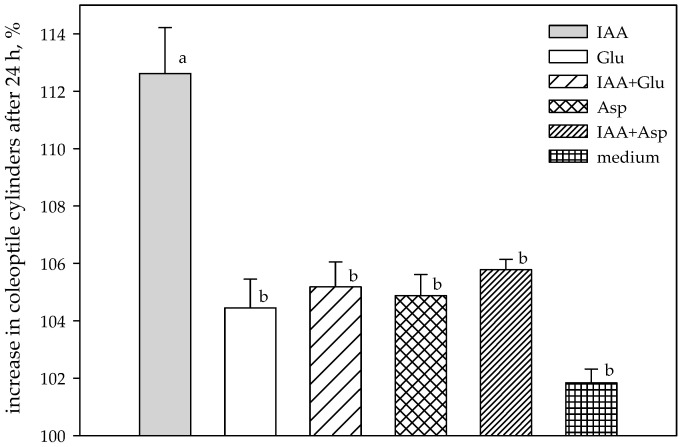
Growth of wheat coleoptile segments treated with exogenous IAA and its conjugates. IAA = 30 µg∙mL^−1^; IAA + Glu = 30 μg∙mL^−1^; IAA + Asp = 30 µg∙mL^−1^; Glu = 30 µg∙mL^−1^; Asp = 30 µg∙mL^−1^; medium (control) = medium alone. Data are means of five replicates. Error bar at each point indicates ± SE. Different letters above each bar indicate significant differences (*p* < 0.001).

At the beginning of the experiment (24 h), the growth of wheat coleoptile segments treated with bacterial IAA was stimulated in a range from 1.98% (HLB 2-6) to 18.72% (KC 1-4). In general, two strains (*Paenibacillus* sp.—PC 2-5 isolated from Hondia coleoptile and *Lysinibacillus* sp.—KC 1-4 originating from Hondia roots) responded to the stimulation in a similar way in the subsequent hours of the experiment (72 and 96 h), when the stimulation of the growth of wheat coleoptile segments oscillated in the range of 7.52–19.98% and 4.60–6.44% at 72 h and 96 h, respectively (Table 2). Other strains responded with growth inhibition in the range of 0.83–2.86% (72 h) and 1.04–2.10% (96 h) in comparison to the control conditions. Conversely, the biological activity of IAA (measured by stimulation of the elongation growth of the coleoptile fragments) synthesized by the root endophytes was higher, likewise IAA produced by the coleoptile-associated endophytes. A similar trend in the high biological activity of IAA was observed for bacterial endophytes isolated from wheat coleoptiles (Table 2). In addition to the free IAA, we also used the biological assay to analyze the artificial conjugates of IAA, which reduce the activity of the hormone. The activity of aspartic acid (Asp) and glucose (Glu) conjugates was tested with the biotest by measuring their effect on coleoptile elongation as described in the Materials and Methods (Section 4.5) and compared to the activity of free IAA. Generally, the conjugates had lower (by 5.35–7.14%) biological activity in comparison to free IAA alone (Figure 3).

### 2.5. Comparison of the Three Methods for Determination of IRCs

The summary of the tests performed with the three independent methods is presented in Table 3. The amounts of IRCs determined with the colorimetric method (Salkowski assay) ranged from 7.256 to 33.298 µg∙mL^−1^. Unfortunately, the Salkowski assay is not capable of distinguishing which IRC is secreted by bacteria. It has been shown that different IRCs can react with the Salkowski reagent and cause color change, e.g., IAA, indole-3-pyruvic acid, and indole-3-acetamide to pink, indole-3-butyric acid to orange, indoxyl sulfate to purple, and indole to brown [20,21].

Thus, to confirm the free IAA production, we used the LC-MS/MS method. The results indicated that the amounts of free IAA constituted from 1.75 to 52.68% of all IRCs and suggested that *Paenibacillus* strain PC 2-5 was the most efficient free IAA producer. In turn, the colorimetric method suggested that *Lysinibacillus* strain KC 1-4 produced higher amounts of free IAA. Furthermore, LC-MS/MS allowed quantitation of free IAA in samples in which there was no color change after the addition of the Salkowski reagent. All these observations confirm that LC-MS/MS is more selective and sensitive (thanks to the capability to separate sample components on a column and the application of the MRM mode) than the colorimetric method. Furthermore, LC-MS/MS can provide complementary information about the production of indole compounds by bacteria that cannot be detected by the Salkowski reagent (e.g., indole-3-lactic acid) [20]. Therefore, the LC-MS/MS approach gives additional information about low-producing strains. To confirm the presence of free IAA among the quantified IRCs, it is advisable to use a simple auxin-specific biotest. The assay determines the level of growth-promoting IAA via the effect on the growth of coleoptile segments.

## 3. Discussion

There are many reports on plant growth-promoting rhizobacteria (PGPR) strains isolated from wheat [22,23,24]. Isolation of PGPR strains is a crucial step in creation of an effective biofertilizer by selection of strains of microorganisms suitable for plants. It has been reported that strains operating in one region of the country/world may not be useful in another region [25]. Moreover, the strains should match the properties of the soil in which they are to be applied. The situation is similar to adjustment of the appropriate medium to cultures of certain microorganisms. Plant growth-promoting endophytes (PGPE) are a very interesting group of microorganisms. A special relationship between the plant and the microorganism is important for the possibility of colonization of plant tissues by various endophytic strains. These endophytes may occur as obligatory, facultative, and occasional microorganisms [26,27]. As summarized by Chang et al., advanced studies provided evidence for recruitment of the advantageous microorganisms from bulk soil into the rhizosphere probably by crops [28]. A similar situation of recruiting favorable taxa (e.g., producing the appropriate combinations of plant hormones) into plant tissues may take place in the rhizosphere, but it is hampered by the difficulty in overcoming the plant cell wall [27].

We studied endophytic bacteria isolated from selected winter wheat species. Endophytic strains of common and spelt wheat cultivars (*Triticum aestivum* ‘Hondia’ and ‘Tytanika’ as well as *T. spelta* ‘Rokosz’) were isolated. The production of IRCs by these bacteria was detected using the Salkowski reagent and the LC-MS/MS approach. The biological activity of compounds secreted into the medium (especially IAA) was confirmed by the biotest, which is specific to free auxins. The color obtained in the Salkowski reaction (from pink to red) indicated different amounts of auxins produced by the different endophyte strains, production of other indole compounds, or/and formation of IAA conjugates. It seems that the pink-type strains not only produce free IAA but can also produce other compounds, as evidenced by the higher values obtained in the colorimetric test. Previously, Gilbert et al. [20] and Tsukanova et al. [29] suggested that the Salkowski reaction is not specific for auxin, thus one cannot be entirely sure whether the studied strain can indeed synthesize IAA or other biologically active auxins [20,29]. Gilbert et al. analyzed bacterial strains from surface-sterilized duckweed tissues for production of IRCs using the Salkowski colorimetric assay. Using mass spectrometry, they confirmed that various bacterial isolates were able to produce IRCs, e.g., indole-3-acetic acid and indole-3-lactic acid (ILA) [20]. Other literature reports indicated that IAA metabolites might also include oxidation products and conjugates with sugars, cyclitols, and amino acids [30,31]. Our LC-MS/MS results also confirmed the production of indole-related compounds by the endophytes of wheat cultivars. The data of the MRM analysis of the bacterial post-culture media (Figure 2C) revealed the presence of other compounds that share the MRM transitions selected for the determination of free IAA, but their peaks appeared at different retention times. We assume that these compounds are IAA conjugates or other indole-related compounds like ILA [20,30]. However, at this stage of research, we did not attempt to verify the presumed compounds.

The studied endophytic bacteria displayed a varied ability to synthesize IRCs depending on the wheat species, the host organism, and the time of culture. The endophytic strains studied were mostly represented by the *Bacillus* genus (85%). As shown earlier, many researchers used the colorimetric method and noted the ability of *Bacillus* representatives to produce IAA. Vyas and Kaur analyzed endophytic strains isolated from leaves of *Adhatoda vasica*. Among them, strain A1B3 identified as *Bacillus thuringiensis* and strain A1B6 identified as *Bacillus* sp. synthesized 27.700 µg∙mL^−1^ and 19.300 µg∙mL^−1^ of IAA, respectively. These results were obtained after 48 h of incubation with the spectrophotometric method (535 nm) with Trp supplied at a concentration of 1.751 mmol L^−1^ [31]. We present data from the spectrophotometric analysis of the whole IRCs pool, including IAA, and our results should be considered in this respect. The *Bacillus* sp. strains were able to synthesize IRCs in the range of 8.119–32.851 µg∙mL^−1^ (TLC 1-8 and HLC 9, respectively) after 48 h in medium supplemented with 5 mmol L^−1^ Trp. Susilowati et al. demonstrated that *Bacillus aerius*, *Bacillus amyloliquenfaciens*, *Bacillus cereus*, and *Bacillus toyonensis* strains produced IAA in amounts ranging from 13.680 µg∙mL^−1^ for *Bacillus toyonensis* to 25.380 µg∙mL^−1^ for *Bacillus amyloliquenfaciens*. Importantly, these authors used similar culture conditions as in the present study. Furthermore, Susilowati et al. also isolated a strain belonging to the genus *Pantoea* synthesizing IAA at the level of 19.320 µg∙mL^−1^ [32]. Our data indicate that the *Pantoea* TLB 1-1 strain produced IRCs only after 168 h of culturing at a similar level as the *Pantoea* strain described by Susilowati and co-workers—13.833 µg∙mL^−1^. Previously, no positive reaction with Salkowski reagent was detected. In turn, *Pantoea agglomerans* DSM 3493 produced IAA only at the level of 0.670 µg∙mL^−1^ [32]. Ferchichi et al. (2019) obtained several endophytic strains representing the following genera: *Burkholderia*, *Rhizobium*, *Pseudomonas*, *Rahnella*, *Klebsiella*, *Pantoea*, *Enterobacter*, *Bacillus*, and *Paenibacillus* from lupine plants. After 120 h of culturing, all isolates showed the ability to produce IAA in a medium supplemented with Trp (2.5 mmol L^−1^). Among them, *Paenibacillus glycanilyticus* NBRC 16,618 produced 18.890 µg∙mL^−1^ of IAA [33]. This is slightly higher than the amount produced by the *Paenibacillus* PC 2-5 strain in this study (13.625 µg∙mL^−1^ after 120 h of culturing), with half the content of Trp in the medium than in the experiment conducted by Ferchichi and co-workers. Govarthanan et al. obtained two IAA-producing *Paenibacillus* strains. *Paenibacillus* sp. RM isolated from roots of *Tridax procumbens* synthesized IAA at the level of 17.200 µg∙mL^−1^ after 48 h of incubation [34]. Our results revealed that *Paenibacillus* strain PC 2-5 isolated from Hondia coleoptiles synthesized IAA at the level of 20.920 µg∙mL^−1^, while *Paenibacillus* strain TPC 1-12, also originating from Hondia coleoptiles, produced IAA at the level of 5.080 µg∙mL^−1^.

Finally, to have a complete picture, it is important to analyze not only the amount of IRCs, especially IAA, but also their biological activities. Hence, we propose a methodological approach based on three complementary methods: colorimetry, chromatography coupled with spectrometry, and bioactivity assessed by analysis of IRCs generated by different strains of wheat endophytic bacteria.

## 4. Materials and Methods

### 4.1. Plant Material

The strains of endophytic bacteria isolated from the leaves, roots, and coleoptiles of *T. aestivum* L. cv. ’Hondia’, *T. aestivum* L. cv. ’Tytanika’ and *T. spelta* L. cv. ’Rokosz’ were studied. Wheat seedlings were obtained from the fields belonging to the Lublin Agricultural Advisory Center (LAAC) in Końskowola, Poland (51°24′33” N, 22°03′06” E) in BBCH-scale 13. The collected plant samples were placed in sterile plastic boxes and immediately transported (at 4 °C) to the laboratory for microbiological analysis. In the laboratory, the soil was removed from the plants in an ultrasonic bath (Emmi 20 HC, EMAG Technologies^®^, Salach, Germany). After that, the leaves, roots, and coleoptiles were separated. Before isolating endophytic strains, the effectiveness of the sterilization method was determined. The sterility of the plant materials was controlled using indirect (culture) and direct (PCR) methods. The last rinse water of the plant material was used as a template. The samples were surface sterilized by dipping in ethanol (96%) for 1 min, sodium hypochlorite (3%) for 6 min, and ethanol (70%) for 1 min and washed with sterilized distilled water three times.

### 4.2. Isolation and Identification of Endophytes

The sterile leaves, roots, and coleoptiles of *T. aestivum* L. cv. ’Hondia’, *T. aestivum* L. cv. ’Tytanika’ and *T. spelta* L. cv. ’Rokosz’ were aseptically cut, macerated, and homogenized with a sterile mortar and pestle to obtain bacterial endophytes from the plant tissues, which were further plated on nutrient agar plates containing nystatin (50 mg∙mL^−1^). The plates were incubated at 30 °C for 120 h. Morphologically distinct colonies were purified by streak plating on nutrient agar. Bacterial DNA was isolated from liquid endophyte cultures as in Banach et al. [35]. Next, the PCR reaction was performed in a reaction mixture containing 1X Phusion Flash High-Fidelity PCR Master Mix (Thermo Scientific, Waltham, MA, USA), 1 µL of template DNA (in the range from 6.60 to 15,160 µg∙mL^−1^, (Table A2, Appendix A), and sterile double-distilled water (free DNase, EURx, Gdańsk, Poland) in a total volume of 25 µL. Universal eubacterial primers (each 1.0 µM) 27F and 1492R (Table A3, Appendix A, Genomed S.A., Warsaw, Poland) were used. The reaction was carried out in the following conditions: 98 °C for 10 s; 30 cycles of 95 °C for 5 s, 56 °C for 5 s, and 72 °C for 40 s (LABCYCLER, SensoQuest GmbH, Göttingen, Germany). The PCR products were run on agarose gel (1%) and visualized with the use of SimplySafe™ (EURx). The control reactions were performed: Negative—containing only sterile double-distilled water (free DNase, EURx) without a DNA template and positive, in which DNA isolated from *E. coli* DH5α™ (Thermo Scientific) was a template. Then, all PCR products were purified and sent to sequencing (Genomed S.A.). The sequences were analyzed by the web-version of the BLASTN algorithm (NCBI, Bethesda, MD, USA) for identification of the isolates. The identified sequences were deposited in the GenBank (NCBI, http://www.ncbi.nlm.nih.gov, accessed on 12 February 2021) under accession numbers shown in Table A1.

### 4.3. Colorimetric Determination of IAA

Bacterial isolates were cultivated on a liquid medium (pH 7.5) with the addition of tryptophan (1 g∙L^−1^) at 30 °C in the dark. To estimate the production of IRC pools containing IAA, the samples were centrifuged and the supernatants were transferred to a glass tube. A portion of each sample was frozen at −20 °C before LC-MS/analysis MS. Culture supernatants from each of the isolates were mixed with the Salkowski reagent (15 mL of 96% H_2_SO_4_, 25 mL dH_2_O, 0.75 mL of 0.5 mol L^−1^ FeCl_3_) in the ratio of 1:2. The original Salkowski reagent formulation was used, likewise the standard time between the addition of the reagent and reading the absorbance [36]. The incubation was carried out for 30 min in the dark. The presence of pink color indicated the production of IRCs (including IAA) and their optical density (OD) was recorded at 530 nm (BioSpectrophotometer, Eppendorf, Hamburg, Germany). Qualitative analysis of IRCs produced by the endophytic bacteria was carried out after 24, 48, 72, 96, 120, 144, and 168 h of culturing. The total IRC content, especially IAA, was calculated from the equation generated from the standard curve prepared in the range of 0 to 100 micrograms of IAA (Sigma-Aldrich, St. Louis, MO, USA) per milliliter (y = 0.0168x + 0.0896; R^2^ = 0.99).

### 4.4. Detection and Quantification of Free IAA by LC-MS/MS

The presence of free IAA in bacterial supernatants was confirmed by liquid chromatography-tandem mass spectrometry (LC-MS/MS). Samples with the highest and the lowest productivity were chosen and analyzed with the colorimetric test considered as the screening test for LC-MS/MS. For the analysis, a 1290 infinity ultra-high performance liquid chromatograph (Agilent Technologies, Santa Clara, CA, USA) coupled with a 6460 triple quadrupole mass spectrometer (QQQ, Agilent Technologies) was used. It was equipped with an electrospray ion source (Agilent Jet Stream) and operated in the positive ion mode. The chromatographic separation was performed on a Zorbax Eclipse Plus-C18 Rapid resolution HT 2.1 × 50 mm, 1.8 µm column coupled with a Zorbax Eclipse Plus-C18 2.1 × 12.5 mm, 5 µm Narrow Bore Guard Column (both from Agilent Technologies) with a mobile phase containing solvent A—0.1% (*v*/*v*) formic acid (for LC-MS, Sigma-Aldrich) in ultrapure water and solvent B—0.1% (*v*/*v*) formic acid in methanol (hypergrade, Merck, Darmstadt, Germany). The gradient program was as follows: 0–5 min—5% to 95% B; 5–10 min—5% B (column re-equilibration). The column temperature and the mobile phase rate were 25 °C and 0.25 mL/min, respectively. The injection volume was 5 µL. IAA determination was performed with the multiple reaction monitoring (MRM) transitions set for 176.1 > 130.0 (quantifier, fragmentor—100 V; collision energy—14 eV) and 176.1 > 103.0 (qualifier, fragmentor—100 V; collision energy—32 eV). The ionization parameters were as follows: nebulizer—35 psi; gas temperature—300 °C; gas flow—6 L min^−1^; sheath gas temperature—300 °C; sheath gas flow—7 L min^−1^; spray voltage—4000 V. The typical MRM spectra obtained during the LC-MS/MS analysis of the IAA standard are presented in Figure 2. Quantitative analysis of IAA was carried out after 24, 48, 72, 96, 120, 144, and 168 h of culturing the endophytic bacteria using matrix-matched calibration. Instrument control and data acquisition were carried out using Agilent MassHunter Acquisition software v.B.08. The data were analyzed by Agilent MassHunter Quantitative Analysis software v.B.07. For LC-MS/MS analysis, the samples were defrosted at room temperature, vortexed thoroughly, and further processed according to a previously published protocol with a slight modification [37]. An aliquot (50 µL) was transferred to the Eppendorf tube and mixed with 400 µL of cold methanol (hypergrade) for protein removal. After incubation at −20 °C for 30 min, the samples were centrifuged at 14,000×*g* for 15 min at 4 °C (5415R Centrifuge, Eppendorf). Supernatants (400 µL) were placed in glass vials, evaporated gently to dryness (Genevac EZ-2 Elite Personal Evaporator, Genevac Ltd., Ipswich, UK), and reconstituted in 100 µL of 0.1% (*v*/*v*) formic acid in ultrapure water. After centrifugation (14,000× *g* for 15 min at 4 °C), the supernatants were transferred to chromatographic insert vials and immediately analyzed in triplicates using the LC-MS/MS method.

Matrix-matched calibration standard solutions were prepared analogously in liquid medium without Trp, used for culturing bacterial isolates, spiked with a known volume of the IAA standard solution in the range from 0.010 to 1.995 µg mL^−1^. The equation of the calibration plot was expressed as *y* = a*x* + b, where *y* corresponds to the IAA peak area, *a* is the slope, *x* is the analyte concentration (µg mL^−1^), and *b* refers to the intercept. LOD and LOQ were calculated as a standard deviation of intercepts (*n* = 3) divided by the slope of the calibration function and multiplied by 3.3 or 10, respectively. The coefficient of determination (R^2^) ≥ 0.99 was required for linearity estimation. The blank medium (without the IAA spike) was also analyzed and the value obtained was subtracted from each calibration point. If needed, the samples were diluted to fit the linear range of the calibration curve and re-analyzed.

### 4.5. Test of IAA Biological Activity (Biotest)

The wheat seeds were soaked for 24 h in distilled water and were grown on a moist filter paper for 5 days in a thermostat in the dark at 21 °C. The coleoptiles were cut into 5 mm long fragments and rinsed for 1 h in deionized water. Next, the coleoptile samples were transferred into the post-culture medium obtained from the endophyte strains for 24 h. After centrifugation, the medium was subjected to estimation of the IAA concentration using the colorimetric and LC-MS/MS methods, and the biotest was carried out with the use of coleoptile cylinders. The endophyte growth medium (containing 5 mmol L^−1^ L-Trp) served as the control.

To determine the effect of IAA conjugates on the elongation growth, 5-mm sections of the coleoptiles were placed in the medium containing exogenously added IAA (30 µg·mL^−1^), IAA + aspartate (30 µg·mL^−1^), and IAA + glucose (30 µg·mL^−1^). After 24 h, the length of the coleoptile sections was measured and compared to the control (length of coleoptile sections in the medium alone). In the case of both the best and poorest IAA-producing bacteria, the effects of IAA extracts were also examined in plant bioassays. Statistical analysis was done using the multiple comparison test (one-way ANOVA). The statistical analyses were performed by means of Statistica 10 software (StatSoft, Tulsa, OK, USA).

## 5. Conclusions

The endophytic bacteria isolated from different tissues of the tested wheat cultivars were able to produce IAA in in vitro conditions via the tryptophan-dependent pathway, which constituted from 1.75 to 52.68% of all IRCs. The LC-MS/MS technique evidenced that *Paenibacillus* originating from Hondia coleoptiles is the most efficient free IAA producer. Concurrently, the results of the colorimetric method suggested that *Lysinibacillus* isolated from Hondia roots should be treated as an efficient free IAA producer. LC-MS/MS was capable of quantitation of free IAA in samples where no color change was recorded by the colorimetric test. This proves that LC-MS/MS is more selective and sensitive than the colorimetric method. In some cases, the bioassay results indicated significant growth of wheat coleoptile segments treated with the bacterial post-culture medium. In this context, it seems reasonable to use the assessed methods in combination to obtain a comprehensive picture of IRC synthesis by different strains of wheat endophytes. To summarize, we have presented a methodological approach in the study of IRC pools by comparison of three methods: colorimetric, LC-MS/MS, and biotest. The results allow formulation of only one main conclusion that the studied post-culture medium contains auxin-type compounds and can support plant development.

## Figures and Tables

**Table 1:** IAA production determined by LC-MS/MS. Abbreviation: *na*—not analyzed. Data are presented as the mean (*n* = 3) ± standard deviation (SD).

Strain Name	Results (µg∙mL^−1^)						
	Time of Cultivation (h)						
	24	48	72	96	120	144	168
HLA 1-7	1.836 ± 0.067	*na*	*na*	*na*	1.810 ± 0.013	1.776 ± 0.018	*na*
HLA 2-7	0.344 ± 0.009	*na*	0.702 ± 0.002	0.668 ± 0.003	*na*	*na*	*na*
HLB 1-3	0.519 ± 0.009	*na*	0.720 ± 0.014	0.686 ± 0.009	*na*	*na*	*na*
HLB 2-6	1.716 ± 0.021	3.958 ± 0.030	4.643 ± 0.045	*na*	*na*	*na*	*na*
K 1-4	0.718 ± 0.013	*na*	*na*	0.731 ± 0.017	0.711 ± 0.014	*na*	*na*
KC 1-4	2.789 ± 0.310	*na*	*na*	*na*	*na*	*na*	6.649 ± 0.032
PC 2-5	11.770 ± 0.094	*na*	*na*	*na*	7.178 ± 0.043	7.181 ± 0.062	*na*
RL 2-2	*na*	0.357 ± 0.006	*na*	*na*	0.301 ± 0.011	0.360 ± 0.013	*na*
RP 2-4	*na*	*na*	0.650 ± 0.004	0.702 ± 0.008	0.500 ± 0.019	*na*	*na*
TLB 2-9	*na*	*na*	0.982 ± 0.002	0.965 ± 0.075	1.207 ± 0.027	*na*	*na*
TPA 1-3	*na*	*na*	2.397 ± 0.038	2.260 ± 0.142	2.226 ± 0.022	*na*	*na*
TKC 2-1	*na*	*na*	*na*	*na*	0.724 ± 0.013	0.558 ± 0.024	0.557 ± 0.006

**Table 2:** Bioactivity of crude bacterial supernatants containing IRCs as % of the control medium (*n* = 5, ±standard deviation, SD). The growth medium of endophytes (containing Trp) was the control for biological activities.

Probes	The Growth of Wheat Coleoptile Segments Treated with Post-Culture Medium [% of Control] Time of Cultivation (h)			
	24	48	72	96
Control	101.998 ± 1.718	113.164 ± 1.779	105.158 ± 1.365	108.532 ± 1.142
HLA 1-7	106.111 ± 2.110	101.622 ± 0.957	102.145 ± 1.365	107.46 ± 1.083
HLA 2-7	105.682 ± 1.688	101.622 ± 0.957	104.283 ± 1.079	106.740 ± 0.993
KC 1-4	117.941 ± 1.077	121.452 ± 1.304	126.178 ± 0.981	113.860 ± 1.051
PC 2-5	121.086 ± 1.802	119.046 ± 487	113.067 ± 0.872	115.532 ± 1.228
HLB 2-6	104.011 ± 1.215	109.016 ± 1.140	104.770 ± 0.867	106.248 ± 458

**Table 3:** Analysis of IRC production with the colorimetric method, biotest, and liquid chromatography-tandem mass spectrometry determination of IAA from all IRCs. Colorimetric method—µg∙mL^−1^ all IRCs, Biotest -, *na*—not analyzed, (+)—significant result of the biotest, (-)—not significant result of the biotest. LC-MS/MS method—µg∙mL^−1^ IAA from all IRCs, nd—not detected. Data are presented as the mean (*n* = 3).

Strain Name	Methods	Results						
		Time of Cultivation (h)						
		24	48	72	96	120	144	168
HLA 1-7	LC-MS/MS	1.836	*na*	*na*	*na*	1.810	1.776	*na*
	Colorimetric method	14.220	12.673	7.970	7.256	7.286	nd	nd
	Biotest	-	*-*	*-*	*-*	-	*na*	*na*
HLA 2-7	LC-MS/MS	0.344	*na*	0.702	0.668	*na*	*na*	*na*
	Colorimetric method	10.708	21.839	7.792	nd	nd	nd	nd
	Biotest	-	*-*	-	-	-	*na*	*na*
HLB 1-3	LC-MS/MS	0.519	*na*	0.720	0.686	*na*	*na*	*na*
	Colorimetric method	13.536	20.9762	10.232	nd	nd	nd	nd
HLB 2-6	LC-MS/MS	1.716	3.958	4.643	*na*	*na*	*na*	*na*
	Colorimetric method	12.077	10.024	nd	nd	nd	nd	nd
	Biotest	*-*	-	-	-	*na*	*na*	*na*
K 1-4	LC-MS/MS	0.718	*na*	*na*	0.731	0.711	*na*	*na*
	Colorimetric method	nd	12.375	11.899	11.214	nd	nd	nd
KC 1-4	LC-MS/MS	2.789	*na*	*na*	*na*	*na*	*na*	6.649
	Colorimetric method	33.298	25.977	24.607	25.500	22.851	24.012	18.446
	Biotest	+	*+*	*+*	*+*	*na*	*na*	*na*
PC 2-5	LC-MS/MS	11.770	*na*	*na*	*na*	7.178	7.181	*na*
	Colorimetric method	28.327	20.917	15.054	18.506	13.625	nd	nd
	Biotest	+	*+*	*+*	+	+	*na*	*na*
RL 2-2	LC-MS/MS	*na*	0.357	*na*	*na*	0.301	0.360	*na*
	Colorimetric method	nd	nd	18.536	13.893	13.506	nd	nd
RP 2-4	LC-MS/MS	*na*	*na*	0.650	0.702	0.500	*na*	*na*
	Colorimetric method	nd	nd	nd	10.976	nd	nd	nd
TLB 2-9	LC-MS/MS	*na*	*na*	0.982	0.965	1.207	*na*	*na*
	Colorimetric method	15.679	10.202	13.119	nd	12.762	nd	nd
TPA 1-3	LC-MS/MS	*na*	*na*	2.397	2.260	2.226	*na*	*na*
	Colorimetric method	16.512	13.893	21.839	nd	18.506	12.792	15.351
TKC 2-1	LC-MS/MS	*na*	*na*	*na*	*na*	0.724	0.558	0.557
	Colorimetric method	nd	nd	nd	nd	nd	31.869	nd

## Sample Availability

Samples of the compounds IAA are available from the authors.

## Appendix A

**Table A1:** List of endophytes isolated and identified in the studied wheat cultivars.

Bacterial Genus	Culture Collection ID	Plant Part	GenBank Accession Number
*Bacillus* sp.	HLA 1-7	Hondia leaf	MT181071
*Bacillus* sp.	HLA 2-1	Hondia leaf	MT181072
*Serratia* sp.	HLA 2-2	Hondia leaf	MT181073
*Bacillus* sp.	HLA 2-5	Hondia leaf	MT181075
*Bacillus* sp.	HLA 2-7	Hondia leaf	MT181077
*Bacillus* sp.	HLB 1-3	Hondia leaf	MT181080
*Bacillus* sp.	HLB 2-6	Hondia leaf	MT181081
*Bacillus* sp.	HLC 10	Hondia leaf	MT181084
*Serratia* sp.	HLC 2	Hondia leaf	MT181085
*Lysinibacillus* sp.	HLC 4	Hondia leaf	MT181087
*Bacillus* sp.	HLC 7	Hondia leaf	MT734565
*Bacillus* sp.	HLC 8	Hondia leaf	MT181091
*Bacillus* sp.	HLC 9	Hondia leaf	MT181095
*Bacillus* sp.	K 1-2	Rokosz root	MT181089
*Bacillus* sp.	K 1-7	Rokosz root	MT181090
*Bacillus* sp.	K 2-4	Rokosz root	MT181092
*Bacillus* sp.	K 4-3	Rokosz root	MT181094
*Bacillus* sp.	K 1-4	Rokosz root	MT181097
*Bacillus* sp.	K 4-6	Rokosz root	MT181098
*Bacillus* sp.	K 4-7	Rokosz root	MT734566
*Bacillus* sp.	KA 1-1	Hondia root	MT181099
*Serratia* sp.	KA 1-2	Hondia root	MT181100
*Bacillus* sp.	KB 1-6	Hondia root	MT181107
*Lysinibacillus* sp.	KC 1-4	Hondia root	MT181112
*Paenibacillus* sp.	PC 2-5	Hondia coleoptile	MT181137
*Bacillus* sp.	RL 2-2	Rokosz leaf	MT181139
*Bacillus* sp.	RL 4-2	Rokosz leaf	MT181142
*Bacillus* sp.	RP 2-4	Rokosz coleoptile	MT181146
*Bacillus* sp.	RP 4-2	Rokosz coleoptile	MT181150
*Bacillus* sp.	TKB 1-4	Tytanika root	MT181152
*Bacillus* sp.	TKC 1-6	Tytanika root	MT181153
*Bacillus* sp.	TKC 1-7	Tytanika root	MT181154
*Bacillus* sp.	TKC 1-8	Tytanika root	MT181155
*Bacillus* sp.	TKC 2-1	Tytanika root	MT181099
*Bacillus* sp.	TKC 2-2	Tytanika root	MT181088
*Bacillus* sp.	TLA 2-11	Tytanika leaf	MT181156
*Bacillus* sp.	TLA 3-2	Tytanika leaf	MT181157
*Pantoea* sp.	TLB 1-1	Tytanika leaf	MT181158
*Bacillus* sp.	TLB 1-2	Tytanika leaf	MT181159
*Bacillus* sp.	TLB 1-3	Tytanika leaf	MT181160
*Bacillus* sp.	TLB 2-7	Tytanika leaf	MT181161
*Bacillus* sp.	TLB 2-9	Tytanika leaf	MT181162
*Bacillus* sp.	TLC 1-8	Tytanika leaf	MT181163
*Bacillus* sp.	TLC 2-2	Tytanika leaf	MT181164
*Bacillus* sp.	TPA 1-2	Tytanika coleoptile	MT181165
*Bacillus* sp.	TPA 1-3	Tytanika coleoptile	MT181166
*Bacillus* sp.	TPA 2-6	Tytanika coleoptile	MT181167
*Bacillus* sp.	TPA 3-10	Tytanika coleoptile	MT181168
*Bacillus* sp.	TPA 3-8	Tytanika coleoptile	MT181169
*Bacillus* sp.	TPB 1-1	Tytanika coleoptile	MT181170
*Bacillus* sp.	TPB 2-7	Tytanika coleoptile	MT734569
*Bacillus* sp.	TPB 2-8	Tytanika coleoptile	MT181173
*Paenibacillus* sp.	TPC 1-12	Tytanika coleoptile	MT181174
*Bacillus* sp.	TPC 1-2	Tytanika coleoptile	MT181175

**Table A2:** Quality and quantity of DNA used in the PCR reaction. Data are presented as the mean (*n* = 3).

Culture Collection ID	Mean Concentration of DNA (µg·mL^−1^)	SD	Mean A 260:280 Ratio	SD	Mean A 260:230 Ratio	SD
HLA 1-7	1025.00	15.00	2.38	0.03	0.59	0.01
HLA 2-1	9120.00	5.00	2.08	0.01	1.47	0.00
HLA 2-2	355.10	1.31	2.16	0.00	0.51	0.00
HLA 2-5	868.33	7.64	2.65	0.06	0.56	0.01
HLA 2-7	481.67	7.64	2.96	0.16	0.34	0.00
HLB 1-3	2370.00	10.00	2.10	0.02	1.35	0.02
HLB 2-6	343.33	2.89	6.45	1.66	0.24	0.01
HLC 10	41.83	0.31	1.76	0.04	0.43	0.02
HLC 2	748.33	18.93	2.58	0.04	0.51	0.01
HLC 4	96.93	0.15	2.07	0.01	1.35	0.01
HLC 7	71.40	0.26	2.10	0.02	0.15	0.00
HLC 8	1715.00	13.23	1.93	0.04	1.32	0.01
HLC 9	13.30	0.10	2.88	0.13	0.01	0.00
K 1-1	3030.00	5.00	1.81	0.02	1.65	0.01
K 1-7	5763.33	10.41	2.07	0.01	1.92	0.01
K 2-4	6.43	0.15	2.16	0.05	1.53	0.07
K 4-3	1025.17	2.04	2.19	0.00	1.01	0.01
K 1-4	83.50	11.32	2.25	0.02	1.66	0.02
K 4-6	14503.33	595.01	1.54	0.02	0.94	0.01
K 4-7	851.67	24.58	2.28	0.09	0.93	0.02
KA 1-1	2835.00	15.00	2.14	0.02	1.91	0.00
KA 1-2	349.00	37.07	1.63	0.06	0.46	0.02
KB 1-6	676.67	2.89	2.33	0.02	1.02	0.01
KC 1-4	5255.00	620.91	1.65	0.03	0.74	0.02
PC 2-5	6703.33	17.56	1.98	0.01	2.28	0.01
RL 2-2	46.03	6.29	1.98	0.01	1.39	0.01
RL 2-4	2006.67	5.77	1.80	0.01	1.80	0.02
RP 2-4	10273.33	270.02	1.99	0.03	1.93	0.12
RP 4-2	143.37	0.57	1.94	0.02	0.41	0.00
TKB 1-4	1471.67	265.58	1.83	0.02	1.11	0.01
TKC 1-6	262.87	6.81	1.45	0.11	1.21	0.08
TKC 1-7	712.77	5.46	2.01	0.01	0.85	0.01
TKC 1-8	2238.33	17.56	1.76	0.04	1.19	0.02
TKC 2-1	33.00	5.00	1.68	0.13	0.36	0.00
TKC 2-2	226.07	3.66	2.01	0.01	0.32	0.01
TLA 2-11	267.63	4.35	1.35	0.02	1.09	0.02
TLA 3-2	11.80	1.41	3.22	0.40	0.55	0.04
TLB 1-1	388.77	11.46	1.99	0.03	0.51	0.01
TLB 1-2	209.80	0.72	1.54	0.01	0.47	0.01
TLB 1-3	862.40	1.49	1.98	0.01	1.23	0.01
TLB 2-7	8923.33	12.58	1.90	0.01	1.89	0.01
TLB 2-9	3908.33	5.77	1.90	0.01	1.57	0.01
TLC 1-8	1383.33	89.63	1.49	0.02	0.46	0.01
TLC 2-2	1835.00	10.00	1.83	0.01	0.81	0.01
TPA 1-2	27.17	2.28	1.61	0.02	0.74	0.04
TPA 1-3	1214.97	2.37	2.02	0.01	1.75	0.01
TPA 2-6	427.73	34.40	1.67	0.07	0.82	0.06
TPA 3-10	374.47	8.12	2.03	0.02	0.71	0.01
TPA 3-8	2078.33	10.41	1.82	0.03	0.98	0.01
TPB 1-1	80.53	0.25	1.92	0.01	0.11	0.00
TPB 2-7	243.83	6.17	1.97	0.02	0.31	0.01
TPB 2-8	236.00	7.21	1.58	0.01	0.39	0.00
TPC 1-12	879.23	10.06	1.97	0.00	1.05	0.01
TPC 1-2	4595.00	21.79	1.79	0.00	1.68	0.01

**Table A3:** Primers used in the studies.

Primer [5′-3′]		References
27F	AGAGTTTGATCATGGCTCAG	[38]
1942R	TACCTTGTTACGACTT	[39]
